# Association of Severe Retinopathy of Prematurity and Bronchopulmonary Dysplasia with Adverse Neurodevelopmental Outcomes in Preterm Infants without Severe Brain Injury

**DOI:** 10.3390/brainsci11060699

**Published:** 2021-05-26

**Authors:** Seong Phil Bae, Seung Han Shin, Young Mi Yoon, Ee-Kyung Kim, Han-Suk Kim

**Affiliations:** 1 Department of Pediatrics, Soonchunhyang University Seoul Hospital, Soonchunhyang University School of Medicine, Seoul 04401, Korea; bsp328@hanmail.net; 2 Department of Pediatrics, Seoul National University Children’s Hospital, Seoul National University College of Medicine, Seoul 03080, Korea; kimek@snu.ac.kr (E.-K.K.); kimhans@snu.ac.kr (H.-S.K.); 3 Department of Pediatrics, Jeju National University Hospital, Jeju University School of Medicine, Jeju 63241, Korea; yoonmiya81@gmail.com

**Keywords:** preterm infants, bronchopulmonary dysplasia, retinopathy of prematurity, brain injury, neurodevelopment

## Abstract

Although impaired neurodevelopment is strongly associated with severe brain injury, most preterm infants survive without severe brain injury. In this study, the association of impaired neurodevelopment and neonatal morbidities of preterm infants was assessed after excluding those with severe brain injury. This was a retrospective study of very low birthweight infants in a single tertiary center. After excluding infants with severe brain injury, the study population was categorized as infants without intraventricular hemorrhage (IVH) and with low-grade IVH. Neurodevelopmental outcomes at a corrected age (CA) of 18–24 months were evaluated using the Bayley Scales of Infant and Toddler Development 3rd Edition (Bayley-III). Cerebral palsy (CP), hearing impairment and blindness were also assessed and compared. Of 240 infants, 25 (11.6%) infants had combined neurodevelopmental impairment (NDI). In the multivariate analysis for combined NDI, small for gestational age (SGA) (adjusted OR 6.820, 95% confidence intervals (CI) 1.770–26.307), moderate to severe bronchopulmonary dysplasia (BPD) (aOR 3.21, 95% CI 1.032–9.999) and severe retinopathy of prematurity (ROP) (aOR 5.669, 95% CI 1.132–28.396) were associated with combined NDI. Among neonatal morbidities, moderate to severe BPD and severe ROP were associated with adverse neurodevelopmental outcomes in preterm infants without severe brain injury.

## 1. Introduction

Severe brain injury, such as high-grade intraventricular hemorrhage (IVH) and periventricular leukomalacia (PVL), is the most important determinant of adverse neurodevelopmental outcomes in preterm infants [1,2]. However, only 9.4 ~ 13% of very preterm infants experience such brain injury during the neonatal period, while most preterm infants have no brain injury or only mild brain injury, such as low-grade IVH [3,4,5]. Given an impaired neurodevelopment rate of 5 ~ 15%, even in preterm infants without severe brain injury, it is necessary to identify predictors for adverse neurodevelopmental outcomes in this population [1,6,7].

Apart from severe brain injuries in preterm infants, the role of low-grade IVH in neurodevelopment has been controversial. One of the largest cohort studies of preterm infants showed that the incidence of cerebral palsy (CP) at two years of age, in infants with low-grade IVH, was higher than that in infants without any IVH [8]. In a single-center study of ELBW infants, the low-grade IVH group had better neurodevelopmental outcomes at 20 months than the no IVH group [9]. However, some studies have reported that low-grade IVH did not increase the risk of adverse neurodevelopmental outcomes [10,11].

Alternatively, other neonatal morbidities in preterm infants have been reportedly associated with adverse outcomes in short-term and long-term periods. Sepsis and necrotizing enterocolitis (NEC) were associated with adverse neurodevelopmental outcomes, which might be mediated by the degree of white matter injuries [12,13]. Bronchopulmonary dysplasia (BPD) and a longer duration of invasive ventilation were associated with growth retardation and impaired neurodevelopment [14,15]. Adverse neurodevelopment was frequently found in preterm infants with retinopathy of prematurity (ROP), especially with severe disease, with or without visual impairment [16,17]. However, these associations have been demonstrated in preterm infants, including populations of preterm infants with or without severe brain injuries. As infants with those morbidities were more likely to have brain injuries, which are highly associated with adverse neurodevelopment, it is worthwhile to investigate the association of neonatal morbidities with neurodevelopmental outcomes after excluding severe brain injuries in preterm infants.

The aim of the current study was to evaluate whether adverse neurodevelopmental outcomes are associated with low-grade IVH compared with no IVH, and to evaluate the associations of other neonatal morbidities, such as sepsis, NEC, BPD and ROP, with neurodevelopmental impairment (NDI) in very low birth weight (VLBW) infants without severe brain injury.

## 2. Materials and Methods

Preterm infants born between January 2015 and December 2017 with a birthweight of less than 1500 g were enrolled in the study. Infants who had major congenital anomalies or severe brain injuries, such as high-grade IVH and PVL, were excluded from the study population. Those who died or were transferred to other hospitals before discharge and those with missing medical records were excluded from the study population. The study population was categorized as the no IVH group and low IVH group, which was diagnosed with brain sonography (USG) and/or brain magnetic resonance imaging (MRI).

Small for gestational age corresponds to birth weight below the 3rd percentile for gestational age at birth according to INTERGROWTH-21st standard [18]. Bronchopulmonary dysplasia (BPD) was defined when respiratory support and/or oxygen supplementation was required at 36 weeks of postmenstrual age (PMA) [19]. Treated patent ductus arteriosus (PDA) was defined as symptomatic PDA requiring medical or surgical treatment. Retinopathy of prematurity (ROP) was staged according to the International Classification of ROP, and severe ROP was defined as those with a treatment threshold in stage III or higher, or in rapidly progressive disease requiring laser photocoagulation [20]. Necrotizing enterocolitis (NEC) was defined as stage II or higher, according to Bell’s Staging Criteria [21]. Experienced pediatric radiologists performed brain USG using Papile’s classification to determine the grade of IVH [22], and grades I or II were defined as low-grade IVH, while grade III or more was defined as high-grade IVH. A brain magnetic MRI at term-equivalent age was performed if significant brain injury was suspected or the birth weight was less than 1000 g.

The Bayley Scales of Infant and Toddler Development 3rd Edition (Bayley-III) results at a CA of 18–24 months were reviewed for each group to evaluate neurodevelopmental outcomes. On the Bayley-III, scores of less than 85 (<−1 SD) in both the cognitive and language domains, or a motor score of less than 85, were defined as developmental delay [23]. Hearing impairment was defined as the need for unilateral or bilateral hearing aids. Infants who met any of the following criteria at a CA of 18–24 months were defined as having combined NDI and were included in the NDI group: delay in Bayley-III, cerebral palsy (CP), hearing impairment or blindness [24]. This study was approved by the Institutional Review Board of Seoul National University Hospital (IRB No. 1511-006-713). The need for informed consent was also waived by the Institutional Review Board of Seoul National University Hospital, and methods were carried out in accordance with relevant guidelines and regulations.

Statistical analyses were performed using Predictive Analytics SoftWare Statistics (v21.0; IBM SPSS Statistics, IBM Corporation, Armonk, NY, USA). To analyze the population characteristics, categorical data were assessed using the chi-squared test and Fisher’s exact test. Continuous data were analyzed by Student’s *t*-test and the Wilcoxon rank-sum test. Neonatal morbidities such as RDS, treated PDA, moderate to severe BPD, severe ROP, NEC and sepsis, as well as gestational age and small for gestational age, were subjected to multivariate logistic regression analysis for combined NDI. To treat multicollinearity, the variance inflation factor (VIF) was calculated, and covariates of VIF > 10 were not included in the multivariate analysis. Goodness of fit was evaluated by the Hosmer–Lemeshow test. Multivariate logistic regression was performed after excluding infants who were not tested by Bayley-III at follow-up as a sensitivity analysis and is provided in the supplementary materials.

## 3. Results

**Figure 1:**
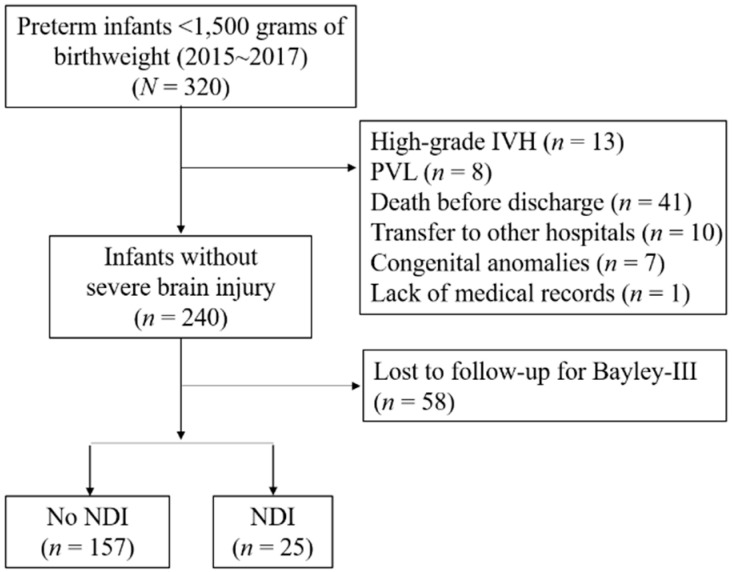
Flow chart of study population. IVH, intraventricular hemorrhage; PVL, periventricular hemorrhage; Bayley-III, Bayley Scales of Infant and Toddler Development 3rd Edition; NDI, neurodevelopment impairment.

Of the 320 VLBW infants, 21 infants with severe brain injury, 41 infants who died in the NICU, 10 infants who were transferred to other hospitals and 7 infants who had major congenital anomalies were excluded from the study (Figure 1). Among the study population, 45 (18.8%) had low-grade IVH, and 25 (11.6%) infants had combined NDI at follow-up.

Gestational age (30.3 vs. 28.4 weeks, *p* < 0.001) and birthweight (1230 vs. 920 g, *p* < 0.001) were lower in the low-grade IVH group (Table 1).

Otherwise, there were no significant differences in the perinatal characteristics. RDS was more prevalent in the low-grade IVH group (41.0% vs. 71.1%, *p* < 0.001), and days of hospitalization were longer in the low-grade IVH group (53 vs. 73 days, <0.001) (Table 2). Other neonatal morbidities were comparable between the two groups.

Combined neurodevelopmental impairment was comparable between the two groups (Table 3).

There was only one case of CP, although a brain MRI at term equivalent age showed no additional injuries, such as cerebellar hemorrhage or white matter injury. There was no case of hearing loss requiring hearing aids, and there was no case of blindness. In Bayley-III at CA 18–24 months, scores of the language domain were lower in the low-grade IVH group, while scores of the cognitive and motor domains were comparable between groups. However, according to the predefined category in developmental delay, there were no differences in the delay of cognitive and language domains, or motor domains, between the two groups. There was no difference in combined NDI between the two groups.

In the univariate analysis, SGA, moderate to severe BPD, severe ROP and sepsis were associated with combined NDI in the study population (Table 4). Multivariate analysis showed that SGA (adjusted OR (aOR) 6.820, 95% confidence intervals (CI) 1.770–26.307), moderate to severe BPD (aOR 3.21, 95% CI 1.032–9.999) and severe ROP (aOR 5.669, 95% CI 1.132–28.396) were associated with combined NDI (goodness of fit, *p* = 0.381).

Among 16 infants with severe ROP, there were no cases of blindness of any eye. Only one infant was prescribed eyeglasses, owing to myopia of both eyes, and had no combined NDI at follow-up. After excluding 58 infants without Bayley-III at follow-up, multivariate analysis also showed that SGA and severe ROP were associated with combined NDI (goodness of fit, *p* = 0.576) (Table S2).

## 4. Discussion

In the current study, 11.6% of VLBW infants without severe brain injury experienced NDI at CA 18–24 months, and there were no differences in NDI between the low-grade IVH group and the no IVH group. Among other neonatal morbidities, SGA, moderate to severe BPD and severe ROP were associated with NDI, although no case of blindness was noted in the study sample. The prevalence of NDI among preterm infants without severe brain injury was comparable with previous studies, as Bolisetty et al. reported that moderate to severe NDI occurred in 13.5% of extremely preterm infants [1], and Payne et al. reported that moderate to severe developmental impairment was found in 10 ~ 30% of extremely preterm infants without high-grade IVH [25].

The association of SGA and adverse neurodevelopment has been well demonstrated in term infants [26,27]. Recently, studies also reported that birth as SGA or intrauterine growth restriction was associated with growth restriction of the brain and adverse neurodevelopment in preterm infants [28,29]. The association of BPD developmental impairment has also been well demonstrated [30,31,32]. Schmidt et al. suggested that BPD affects later death or disability at five years of age, with an odds ratio similar to that of brain injury [33].

Retinopathy of prematurity (ROP) is frequently found in preterm infants with low gestational ages and is associated with adverse neurodevelopmental outcomes, especially in severe diseases [16,33]. However, severe ROP is also associated with the presence of severe IVH [34,35]. Given that several conditions in preterm infants, such as tissue ischemia, vascular immaturity and oxidative stress, are associated with these two conditions [36,37], studies reporting the association between severe ROP and NDI included up to 10 ~ 50% infants with severe brain injury in the study population [16,33,38]. Therefore, excluding those with severe brain injury might be helpful to clarify the association between severe ROP and NDI more clearly, as shown in this study.

One of the hypotheses to explain the association between neurodevelopment and ROP is that visual impairment might mediate neurodevelopmental impairment (NDI) in preterm infants with severe ROP [39,40,41]. Early childhood sensory experience after brain damage affects the reorganization of synapses through neuroplasticity and thereby affects high-order cognitive function [42,43]. However, in the current study, there were no infants with blindness or deafness. Only one infant was prescribed eyeglasses, but the infant had no NDI at follow-up. Rather, the association between severe ROP and nonvisual disabilities might originate from unfavorable exposures that promote the development of retinopathy and may simultaneously have a negative effect on brain plasticity [17]. In studies with brain MRI at term-equivalent age, severe ROP was associated with a delay in white matter maturation and reduced brain volume [16,41].

Another interesting finding is that low-grade IVH was not associated with neurodevelopmental impairment in this study. There have been studies reporting the association of low-grade IVH with neurodevelopmental outcomes [7,9]. However, Payne et al. reported that the neurodevelopmental outcomes at 18 to 22 months CA of preterm infants who were born at less than 27 weeks of gestation with low-grade IVH were not different from those without hemorrhage. A report from de Vries et al. also illustrated comparable outcomes between the low-grade IVH group and the control group [6]. Those studies regarding the association of low-grade IVH and neurodevelopmental outcomes did not consider ROP as a cofounding factor. Only the study by Klebermass-Schrehof et al. reported the prevalence of severe ROP between low-grade IVH and no IVH, but severe ROP was not considered when comparing outcomes [7]. The present study investigated the impact of low-grade IVH, as well as severe ROP, and showed no association of low-grade IVH and neurodevelopment outcomes. However, the results of the current study should be interpreted with caution, as the incidence of low-grade IVH among the study population without severe brain injury was relatively lower (18.8%) than previous studies (26 ~ 40%), with relatively low incidence of NDI [1,7,9,25]. This might be attributed by different study populations, as more mature preterm infants were included in the current study. Although there was a discrepancy of the number of participants between groups, incidence of low-grade IVH was adjusted with other factors to calculate adjusted odds for NDI as a study outcome.

There are several limitations to this study. First, among 240 VLBW infants without severe brain injury, only 182 (75.8%) were evaluated by Bayley-III. When infants without Bayley-III scores were excluded as a sensitivity analysis, multiple logistic regression analysis found that severe ROP and SGA were still associated with NDI (Table S2). Second, data were not available on socioenvironmental influences, such as the education level and socioeconomic status of the parents.

## 5. Conclusions

Among VLBW infants without severe brain injury, 11.6% had NDI at CA 18–24 months. Severe ROP with a treatment threshold and moderate to severe BPD were associated with NDI. However, no case of blindness was identified. As severe brain injury, as a strong predictor of NDI, was excluded in the study population, the role of ROP and BPD in neurodevelopment might be clearer in this study.

## Figures and Tables

**Table 1:** Perinatal characteristics of study population.

	No IVH (*n* = 195)	Low-Grade IVH (*n* = 45)	*p*-Value
GA (week)	30.3 (28.3–32)	28.4 (26.4–30.4)	<0.001
Birth weight (g)	1230 (980–1340)	920 (680–1210)	<0.001
SGA	49 (25.4)	12 (27.3)	0.849
IVF	95 (48.7)	21 (46.7)	0.869
Male	98 (50.3)	26 (57.8)	0.410
C/S	120 (61.5)	30 (66.7)	0.609
hCAM	68 (36)	22 (51.2)	0.083
PPROM >18 h	52 (27.8)	19 (43.2)	0.068
Oligohydramnios	36 (20.2)	13 (31)	0.150
PIH	26 (13.6)	9 (20.9)	0.239
No ACS	34 (17.7)	6 (13.6)	0.516

Data are shown as the *n* (%) or median (interquartile range). Abbreviations: ACS, antenatal steroid; C/S, cesarean section; GA, gestational age; hCAM, histologic chorioamnionitis; IVF, in vitro fertilization; *n*, number in group; PIH, pregnancy-induced hypertension; PPROM, preterm premature rupture of membrane; SGA, small for gestational age.

**Table 2:** Neonatal morbidities of study population.

	No IVH (*n* = 195)	Low-Grade IVH (*n* = 45)	*p*-Value
RDS	80 (41)	32 (71.1)	<0.001
Treated PDA	52 (26.7)	20 (44.4)	0.029
Moderate to severe BPD	51 (26.6)	18 (40)	0.074
Any stage ROP	40 (20.7)	17 (37.8)	0.016
Severe ROP	10 (5.1)	6 (13.3)	0.088
NEC	7 (3.6)	3 (6.7)	0.403
Sepsis	9 (4.6)	4 (8.9)	0.273
Hospital stay (days)	53 (32–79)	73 (55–95.5)	<0.001

Data are shown as the *n* (%) or median (interquartile range). Abbreviations: BPD, bronchopulmonary dysplasia; IVH, intraventricular hemorrhage; *n*, number in group; NEC, necrotizing enterocolitis; PDA, patent ductus arteriosus; RDS, respiratory distress syndrome; ROP, retinopathy of prematurity.

**Table 3:** Neurodevelopmental outcomes at 18–24 months of corrected age.

	No IVH (*n* = 195)	Low-Grade IVH (*n* = 45)	*p*-Value
Neurodevelopmental impairment	19 (9.7)	6 (13.3)	0.430
Cerebral palsy	1 (0.5)	0 (0)	1.000
Hearing loss	-	-	-
Blindness	-	-	-
Bayley-III	(*n* = 146)	(*n* = 36)	
Cognitive domain	100 (90–105)	90 (87.5–110)	0.484
Language domain	97 (86–106)	90 (79–100)	0.025
Motor domain	97 (88–103)	97 (88–100)	0.617
Cognitive and language <85	11 (7.5)	3 (8.3)	1.000
Motor <85	15 (10.3)	6 (16.7)	0.380

Data are shown as the *n* (%) or median (interquartile range). Abbreviations: Bayley-III, Bayley Scales of Infant and Toddler Development-Third edition; IVH, intraventricular hemorrhage.

**Table 4:** Univariate and multivariate analysis for combined neurodevelopmental impairment at 18–24 months of corrected age.

	Univariate Analysis			Multivariate Analysis			
	OR	95% CI	*p*-Value	Adjusted OR	95% CI	*p*-Value	VIF
GA (week)	0.90	(0.77–1.04)	0.141	1.00	(0.75–1.33)	0.986	2.830
SGA	3.07	(1.32–7.16)	0.009	6.82	(1.77–26.31)	0.005	2.670
RDS	1.82	(0.78–4.24)	0.163	2.44	(0.57–10.44)	0.228	1.810
Treated PDA	1.65	(0.70–3.86)	0.253	0.87	(0.29–2.67)	0.814	1.810
Moderate to severe BPD	5.33	(2.23–12.78)	0.000	3.21	(1.03–10.0)	0.044	1.680
NEC	4.05	(0.98–16.80)	0.054	1.94	(0.34–11.08)	0.457	1.330
Severe ROP	8.90	(2.97–26.71)	0.000	5.67	(1.13–28.40)	0.035	1.230
Sepsis	4.36	(1.24–15.38)	0.022	3.71	(0.72–19.03)	0.116	1.120
Low-grade IVH	1.43	(0.53–3.80)	0.479	0.71	(0.21–2.46)	0.594	1.080

Abbreviations: BPD, bronchopulmonary dysplasia; CI, confidence interval; GA, gestational age; IVH, intraventricular hemorrhage; *n*, number in group; NEC, necrotizing enterocolitis; OR, odds ratio; PDA, patent ductus arteriosus; RDS, respiratory distress syndrome; ROP, retinopathy of prematurity; SGA, small for gestational age; VIF, variance inflation factor.

## Data Availability

The data presented in this study are available on request from the corresponding author. The data are not publicly available due to personal medical records.

## Supplementary Materials

The following are available online at https://www.mdpi.com/article/10.3390/brainsci11060699/s1, Table S1: Perinatal and neonatal characteristics of study population after excluding infants without Bayley-III; Table S2: Univariate and multivariate analysis for combined neurodevelopmental impairment after excluding infants who were not tested with Bayley-III at 18–24 months of corrected age.
