# Supplementary material for: Association of Severe Retinopathy of Prematurity and Bronchopulmonary Dysplasia with Adverse Neurodevelopmental Outcomes in Preterm Infants without Severe Brain Injury
Source: Brain Sci. 2021 May 26;11(6):699. doi: 10.3390/brainsci11060699 (PMC8226991; doi:10.3390/brainsci11060699)
Supplement: Supplementary file 1 [file brainsci-11-00699-s001.zip › brainsci-1208318-supplementary.pdf]

Supplementary materials

Association of severe retinopathy of prematurity and bronchopulmonary dysplasia with adverse neurodevelopmental outcomes in preterm infants without severe brain injury

Authors: Seong Phil Bae[1], Seung Han Shin[2], Young Mi Yoon[3] Ee-Kyung Kim[2] and Han-Suk Kim[2]

[1] Department of Pediatrics, Soonchunhyang University Seoul Hospital, Soonchunhyang University School of Medicine, Seoul 04401, Korea; bsp328@hanmail.net
[2] Department of Pediatrics, Seoul National University Children's Hospital, Seoul National University College of Medicine, Seoul 03080, Korea; kimek@snu.ac.kr (E.-K.K.); kimhans@snu.ac.kr (H.-S.K.)
[3] Department of Pediatrics, Jeju National University Hospital, Jeju University School of Medicine; Jeju 63241, Korea; yoonmiya81@gmail.com
* Correspondence: revival421@snu.ac.kr; Tel.: +82-2-2072-7230

Table S1. Perinatal and neonatal characteristics of study population after excluding infants without Bayley-III

	**No IVH (n=146)**	**Low-grade IVH (n=36)**	p-value
GA (week)	30 (28–31.6)	28.4 (26.8–30.3)	<0.001
Birth weight (g)	1195 (940–1340)	945 (670–1225)	<0.001
SGA	45 (31.3)	11 (31.4)	1.000
IVF	67 (45.9)	16 (44.4)	1.000
Male	68 (46.6)	20 (55.6)	0.357
C/S	91 (62.3)	24 (66.7)	0.702
hCAM	57 (40.1)	17 (50)	0.336
PPROM >18 hr	44 (31.7)	17 (48.6)	0.075
Oligohydramnios	31 (23.1)	10 (30.3)	0.377
PIH	21 (14.7)	8 (22.9)	0.305
Antenatal steroid	64 (43.8)	19 (52.8)	0.356
RDS	63 (43.2)	26 (72.2)	0.003
Treated PDA	42 (28.8)	16 (44.4)	0.029
Moderate to severe BPD	44 (30.3)	15 (41.7)	0.234
Severe ROP	10 (6.9)	5 (13.9)	0.181
NEC	6 (4.1)	3 (8.3)	0.384
Sepsis	8 (5.5)	4 (11.1)	0.258
Hospital stay (days)	59 (35–83)	74.5 (56.5–96.5)	0.007

Data are shown as the n (%) or median (interquartile range). Abbreviations: BPD, bronchopulmonary dysplasia; C/S, cesarean section; GA, gestational age; hCAM, histologic chorioamnionitis; IVF, in vitro fertilization; IVH, intraventricular hemorrhage n, number in group; NEC, necrotizing enterocolitis; PDA, patent ductus arteriosus; PIH, pregnancy induced hypertension; PPROM, preterm premature rupture of membrane; RDS, respiratory distress syndrome; ROP, retinopathy of prematurity; SGA, small for gestational age.

Table S2. Univariate and multivariate analysis for combined neurodevelopmental impairment after excluding infants without Bayley-III.

	**Univariate analysis**			**Multivariate analysis**			
	**OR**	**95% CI**	**p-value**	**adjusted OR**	95% CI	p-value	VIF
GA (week)	0.93	(0.80–1.09)	0.397	1.02	(0.76–1.38)	0.884	2.660
SGA	4.54	(1.86–11.07)	0.001	10.75	(2.53–45.65)	0.001	2.660
RDS	1.68	(0.71–3.97)	0.235	3.09	(0.68–13.97)	0.142	1.970
Treated PDA	1.51	(0.63–3.61)	0.350	1.00	(0.32–3.16)	0.996	1.840
Moderate to severe BPD	4.67	(1.92–11.36)	0.001	2.53	(0.79–8.10)	0.117	1.550
NEC	3.43	(0.80–14.72)	0.097	1.29	(0.21–7.87)	0.781	1.330
Severe ROP	7.24	(2.35–22.34)	0.001	7.62	(1.41–41.28)	0.019	1.250
Sepsis	3.55	(0.98–12.81)	0.053	2.94	(0.54–15.93)	0.212	1.140
Low grade IVH	1.34	(0.49–3.64)	0.569	0.75	(0.21–2.63)	0.657	1.100

Data are shown as the n (%) or median (interquartile range). Abbreviations: BPD, bronchopulmonary dysplasia; CI, confidence interval; GA, gestaionatal age; IVH, intraventricular hemorrhage; n, number in group; NEC, necrotizing enterocolitis; OR, odds ratio; PDA, patent ductus arteriosus; RDS, respiratory distress syndrome; ROP, retinopathy of prematurity; SGA, small for gestational age; VIF, variance inflation factor
